# Modelling Protein Synthesis as A Biomarker in Fragile X Syndrome Patient-Derived Cells

**DOI:** 10.3390/brainsci9030059

**Published:** 2019-03-11

**Authors:** Rakhi Pal, Aditi Bhattacharya

**Affiliations:** Centre for Brain Development and Repair, Institute for Stem Cell Biology and Regenerative Medicine, GKVK Post, Bellary Road, Bengaluru 560065, India; rakhip@instem.res.in

**Keywords:** protein synthesis, Fragile X Syndrome, biomarker, iPSC, fibroblast, lymphoblast

## Abstract

The most conserved molecular phenotype of Fragile X Syndrome (FXS) is aberrant protein synthesis. This has been validated in a variety of experimental model systems from zebrafish to rats, patient-derived lymphoblasts and fibroblasts. With the advent of personalized medicine paradigms, patient-derived cells and their derivatives are gaining more translational importance, not only to model disease in a dish, but also for biomarker discovery. Here we review past and current practices of measuring protein synthesis in FXS, studies in patient derived cells and the inherent challenges in measuring protein synthesis in them to offer usable avenues of modeling this important metabolic metric for further biomarker development.

## 1. Fragile X Syndrome as a Translational Control Disorder—Early Studies Leading to General Consensus

Fragile X Syndrome (FXS) is a triplet repeat disorder, where runaway expansion of CGG repeats at the promoter region of *FMR1* gene causes promoter hypermethylation leading to failure of gene transcription and subsequently loss of protein expression of Fragile X mental retardation protein (FMRP) [1]. FMRP is a versatile protein important in a variety of cellular processes which include ribonucleoparticle packaging and transport [2], mediating micro RNA interactions with target mRNA [3,4], translation elongation [5] and interaction with large conductance ion channels [6]. It is, therefore, not unexpected that the major role of FMRP is in determining timely mRNA translation and by extension, loss of FMRP in FXS, has been noted to cause an imbalance in de novo protein synthesis. 

The above states the general consensus that has emerged in the FXS field, through multiple progressive contributions from varied groups [7]. In the 1990s, FMRP as a constituent of ribonucleoparticle (RNP) and mRNA binding protein was uncovered, following which its role in sensing synaptic activity and coupling it to trafficking of RNPs in dendrites and spines was established [8,9,10]. The association of FMRP with ribosomes was established by multiple studies [11,12,13,14,15,16], while the evidence that FMRP suppresses the translation of its target mRNAs was provided by Laggerbauer et al. [17] and Li et al. [18] in rabbit reticulocyte lysates. Two seminal studies in 2011 [19] and 2014 [20] showed that FMRP actively engages in ribosome stalling and does so by structurally occupying the cleft between ribosomal subunits and mRNA respectively. These findings firmly established the idea of a ‘translational brake’, which received ancillary support from studies that showed FRMP-Cyfip1 association occludes eIF4E from interacting with a productive cap-binding complex and initiating translation [21].

This mechanism of FMRP action led to a natural investigation into the net rates of proteins accumulation in cells lacking FMRP, hence modeling FXS. It was actually in rabbit reticulocytes that loss of FMRP was first reported to cause enhanced protein synthesis [18]. However, almost all work that can be found on this topic is done in neurons and brain slice preparations from FXS mouse models, since it was believed that FMRP expression was largely limited to neurons. Expression profiling in the brain was undertaken by Devys et al., [22] which registered the highest expression of FMRP in cerebellar grey matter in human sections, while Bakker et al., [23] did so in mice tissues. Both studies observed little to no glial staining and focused primarily on neurons. Taminini et al. [24] testing a variety of FMRP antibodies, concluded that majority of FMRP staining was neuronal, giving rise to the notion that FMRP expression is limited to neurons in CNS. It took Wang et al., [25] followed by Pacey and Doering [26], to show the developmental stage specific expression of FMRP in glial cells. It is noteworthy that the initial characterization of the FXS mice model [27] included spine morphology and behavioral experiments. Beebe-Smith and colleagues [28] provided the first metabolic readout in FXS mice, showing elevated cerebral glucose metabolism rates across 38 brain regions. The highest elevation was seen in areas that were active in behaviors such as open field activity and passive avoidance or areas with highest FMRP expression in control animals. This group followed up this work with the first comprehensive study of protein synthesis rates in 2005 [29] using in vivo L-[1-14C] leucine intra-arterial introduction to label nascent proteins within a 60 min time window in four- and six-month-old mice. A total of 75 brain areas were monitored, and overall a 10% increase in basal protein synthesis rates was reported compared to wild type littermates, with differences ranging from 18% in the paraventricular nucleus to 4% in the periaqueductal grey. This study forms an important resource for any proteostatic study in FXS, but it is relevant to note that these were averaged across a brain region and did not distinguish between specific cell types.

Experiments tracking protein synthesis in FXS model mice have largely concentrated on the hippocampus and that too in the context of activation of metabotropic glutamate receptor 5 (mGluR5), [30,31,32]. This focus on one specific brain area arose from data that showed that mGluR activation in area CA1 of the hippocampus induced a protein-synthesis dependent form of long-term depression (LTD) [33] and that this LTD was exaggerated in FXS [34]. Additionally, FMRP was shown to be a conduit of this mGluR5-protein synthesis connection by Muddashetty et al. [35] using mouse synaptoneurosomes and S^35^ methionine labelling of nascent proteins. Hence hippocampal mGluR5-LTD and protein synthesis measures in slices quickly emerged as two experiments that could be used as yardsticks to measure the efficacy of pharmacologic and genetic interventions in mice models over the next several years. The biochemical readout of de novo protein synthesis was facilitated by the development of a method to measure translation in acutely-prepared intact hippocampal slices [30], marrying techniques of electrophysiological slice preparation and incubation with metabolic S^35^ methionine labeling. This technique enjoys widespread acceptance in the FXS community [31,32,36,37]. Measuring protein synthesis rates in vivo, in humans, has been fraught with experimental confounds like anesthetic administration [38]. However, the same group has recently published a method to measure cerebral translation rates in awake subjects as well [39] and hence raises the expectation of doing the same in FXS cohorts. 

With the advent of non-radioactive probes for measuring protein synthesis in the past decade, it has become easier for a variety of labs and clinics to measure protein synthesis. These tools were quickly embraced for studying translation control in FXS. SUnSET or Surface sensing of translation [40] was employed by Bhattacharya et al [41], to check whether a genetic deletion of S6K1 rescued FXS mouse phenotypes. SUnSET employs sub-critical levels of puromycin as a t-RNA analog that end labels nascent proteins in the ribosomes. SUnSET was subsequently used to measure translation in FXS model mice [42,43,44] and human patient derived fibroblasts [45]. Cell-type specific monitoring of translation using non-canonical amino acid tagging (NCAT, [46]) was first employed to measure translation rates in FXS patient derived lymphoblastoid cells (LCLs) [47], following which this technique was adopted for use in acute brain slices [48,49]. This method has two variants BONCAT (Bio-orthogonal non-canonical amino acid tagging) and FUNCAT (fluorescent non-canonical amino acid tagging) which employs orthogonal amino acid substitutes to label newly synthesized proteins, which are then tagged using click-chemistry and then detected by western blot (BONCAT) using biotin or fluorescent-tagged alkynes or azides. It is currently unclear if there is any disruption in astrocytic protein synthesis in FXS.

Taken together, the nexus of protein synthesis, FMRP loss and FXS pathology has been a cornerstone of mechanism-based research in this field. Using multiple measurement techniques, in multiple model systems (mouse, rat, drosophila, zebrafish) it can be shown that there is a surfeit of basal translation in FXS models compared to matched controls [30,36,41,50,51]. Resetting translational homeostasis using a variety of interventions [30,37,41,43,52,53,54,55] have yielded ameliorative effects on a wide range of FXS phenotypes correlated with the human condition. Therefore, measuring protein synthesis and its regulation has become an inescapable benchmark of model validity and/or monitoring treatment outcome efficacy.

## 2. Patient-Derived Models of FXS that Model ‘Disease in a Dish’

Studying neurologic disorders, are amongst the most challenging largely due to inaccessibility of neural tissue. The traditional source has been post mortem samples, which are perhaps more useful in studying pathological changes in ageing [56] and neurotrauma [57]. It is also easier to build a sample repository for these conditions simply due to higher numbers and lifespan ranges of the patients. In contrast it is extremely challenging to retrieve post-mortem tissue for neurodevelopmental disorders like ASDs, intellectual disabilities, spina bifida, fetal alcohol syndrome, etc., [58] owing to younger ages of the patients and far fewer fatalities. Even when post-mortem tissue is available, experimental results are strongly influenced by the manner in which tissue was harvested, preserved, tissue pH, transit time, and duration of storage. Finally, measuring live cell metrics, like metabolism and protein synthesis, is not possible in post-mortem tissues.

Human Embryonic stem cells (huESC) were first isolated by Thomson et al., [59] in 1998 from the inner cell mass of the blastocyst of pre-implantation human embryos. These cells are pluripotent and can be further differentiated to cell types of interest. Verlinsky et al. [60] isolated the first FXS huESCs followed by multiple other groups [61,62,63]. The *FMR1* gene is found to be expressed in pluripotent stem cells but can undergo transcriptional silencing upon differentiation [64]. Over the years, the use of hECS has been mired in ethical issues which has limited the availability of cell lines to researchers. Therefore, the bulk of precision medicine efforts in other diseases, has come to rely on the availability of induced pluripotent technology (iPSC) [65], to generate human iPSCs (hiPSCs) from fibroblast samples. In the FXS field, Urbach et al., [66], first reported the generation of hiPSC from fibroblasts of individuals carrying the FXS mutation followed by others [67,68].

One critical difference between huESC and hiPSC in FXS is that the epigenetic silencing due to hypermethylation of the CGG repeats can be retained in the reprogrammed cells. This naturally spurred researchers to use FXS hiPSCs to study DNA methylation as an in vitro human model of FXS (see Bhattacharyya and Zhao [69], 2016 for an in-depth review). Hierarchical clustering and analysis of DNA microarray show that the hiPSCs cluster together with human ESCs [70] and functionally can form embryoid bodies that develop into the three germ layers besides forming teratomas [71]. Also, the *FMR1* gene is expressed in both differentiated and undifferentiated wild type cells and is transcriptionally silenced in patients [72]. It is worth noting, that Sheridan et al., [67], first reported that reprogrammed FXS fibroblasts showed an instability in the CGG trinucleotide stretch in the 5′ UTR of the *FMR1* gene and some of the FXS hiPSC clones had repeat lengths shorter than the control fibroblast samples. Additionally, in the same study, multiple hiPSC clones were derived from mosaic individuals resulting in a set of genetically matched hiPSC lines (isogenic pairs).There have been no reports of reprogramming human lymphoblastoid cell lines (LCL) from FXS mutant patients. 

Further, studies have investigated the effects of the FXS mutation on iPSC differentiation to a neural lineage. However, none of these reports have been conclusive; possibly due to the genetic differences in samples, source of human material and differentiation protocols used. Castren et al., [73] reported that differentiation of neurospheres derived from post mortem human FXS brain and control fetal brain showed differences in neurite length, number, morphology, and altered Tuj1 to GFAP ratio while Bhattacharyya et al. [74] found no significant difference in neurons differentiated from FXS and healthy samples. Contrastingly, Sheridan et al. [67] demonstrated FXS-associated morphological differences in hiPSC-derived neurons, with FXS cells having fewer and shorter neurites than control cells. Investigating beyond morphological parameters have revealed electrophysiological deficits in huES derived FXS samples. In a series of studies by Ben-Yosef’s group [75,76,77] several functional deficits in FXS neurons differentiated in vitro huESC have been demonstrated. These include the inability to fire repetitive action potentials (AP), reduced AP amplitude and longer AP duration and immature responses to GABA [78]. Therefore, to date a variety of groups have shown different phenotypes in neurons-derived from FXS hiPSCs with more focus on discovery rather than developing a consistent cell-autonomous or network-level biomarker for modeling FXS. 

## 3. Protein Synthesis Studies in Human Derived Cells in FXS

In contrast to the remarkable amount of work done in understanding the methylation and transcriptional silencing, structural and electrophysiological deficits in FXS huESC and hiPSCs, there are but a handful of studies that have measured protein synthesis in these cells. The first study was in two control and patient-derived LCL lines, wherein FUNCAT was used to measure steady-state and IL-2 induced translation [47]. While FXS LCLs showed increased basal protein synthesis, the translation rates decreased in IL-2 stimulated cases contrary to matched controls. A subsequent publication [79], reverted to a more traditional method to test translation in patient-derived fibroblasts using H^3^ leucine autoradiography. This study utilized eight FXS and nine control fibroblast lines spanning a large age range (0–62 years for controls and 1–23 years for FXS). All FXS lines were from patients with greater than 200 repeats, including mosaics. The study reported elevated rates of leucine incorporation in five of the eight FXS lines, with the other three showing comparable translation to WT controls. Grouped data across cell lines did show an extremely significant elevation in protein synthesis rates of patients-versus controls. Curiously, protein synthesis appeared to be correlated with phospho-S6 ribosomal protein abundance in control samples, whereas it had a significant direct correlation with age for FXS lines. This runs counter to Qin (2005) study in mice, where there was a sharp drop in translation across brain areas as soon as FXS model mice attained middle-age. Furthermore, FXS lines showed the same negative correlation with age for phospho-states of mTOR, S6K1, and ERK 1/2, as has been shown in many other cases [80]. These pro-translation kinases have been found to be upregulated in FXS animal models, and targeting these molecules, either genetically or pharmacologically has been found to ameliorate several behavioral, synaptic and morphological phenotypes associated with FXS [41,52]. Therefore the conundrum of decreasing mTOR and S6K1 phosphorylation and hence activation, yet increasing protein synthesis drive remains to be reconciled.

Though the Kumari et al. [79] study had a larger cohort, it was not truly multi-center, that would better simulate a large clinical trial setting. This was addressed by a recent study that combined protein synthesis measurements across three laboratories in Europe and US [45]. The study measured rates of protein synthesis in fibroblasts from 32 individuals with FXS and compared them to 17 controls (ages 6–69 years for FXS; 10-50 years for controls). Further, they compared translation rates in FXS model mouse embryonic fibroblasts and primary neurons within the same experimental parameters. An important finding in this study is the inherent consistency in control fibroblasts across passages, which did not hold for the FXS counterparts. It is not entirely clear if control and FXS cells underwent SUnSET as yoked sets, wherein control and FXS fibroblast samples grown under similar conditions underwent SUnSET puromycin and processing at the same time, which has important implications for the metabolic variability of the samples. A noteworthy finding of this study was that almost a third of the FXS lines had puromycin labeling similar to controls. No correlation with age, mRNA and FMRP protein levels was to be found again. The study further explored the consistency of fibroblasts and neurons from FXS model mice, which has yielded far more consistent elevation in protein synthesis in previous studies [29,31,32,41]. Individual preparations of mouse embryonic fibroblasts (MEFs) and neurons had more variability for the FXS sets and the population spreads were non-normal, however the overall elevation in protein synthesis levels held in neurons and MEFs.

**Figure 1:**
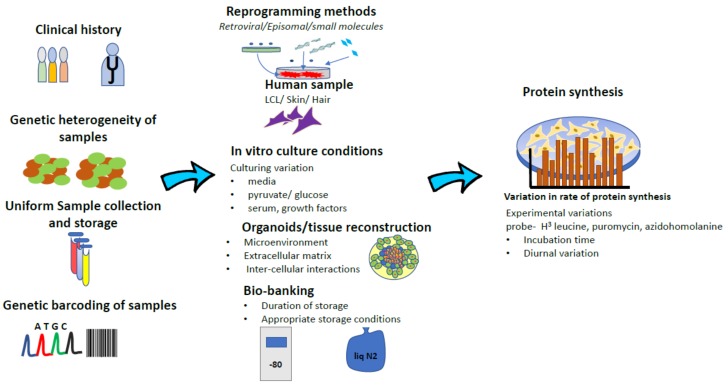
Schematic representation of intrinsic variation at multiple levels (tempo-spatial, tissue, experimental protocols) during development of essential patient derived substrates that influence decisive outcome measures such as protein synthesis.

The disparate results from these studies gives an important insight into the heterogeneity in FXS. Contrary to simple and elegant molecular etiology, FXS is not one disease. While there are stand-alone “FXS-only” patients and there also exist large patient subsets like FXS ± ASD, FXS ± Prader-Willi etc. Therefore, sample and patient stratification is very important at the outset. Similarly, mRNA translation or protein synthesis is a metabolic process that is exquisitely sensitive, to the genetic background, culturing conditions of cells and method of detection used. It would have been useful to know if there are de novo mutations in translation control and executive factors like eEF2, in any of the cell lines that was tested. These likely have a strong bearing on the total efficiency of the translation process that is being studied (see Figure 1 for a graphical depiction). A quick comparison of the culturing conditions in these three studies yields different culturing and experimental conditions in each study. LCLs used in Gross and Bassell [47] used the obligate RPMI media supplemented with FBS, with methionine withdrawal- being the only stressor for the protein synthesis experiment. Kumari et al. [79] fibroblasts were cultured in DMEM + FBS, while the protein synthesis assay was done with cells incubated in ACSF. This incubation starved cells of amino acids and growth factors when the radioligand was added. Jaquemont et al. [45] fibroblasts were cultured in DMEM/F12 + FBS with translation assay done on cells that were serum starved for 16 hours, followed by recovering in FBS for four hours, after with puromycin was added for 30 min. Therefore, it is possible that the outcomes of this metabolic assay could be sensitive to culturing conditions and serum withdrawal states.

## 4. Translation, Neural Differentiation and Loss of FMRP: Challenges for Deploying Translation as a Biomarker

A key property of any deployable biomarker is prior knowledge of its variation and natural history. Variability in translation in fibroblasts has been reported in almost all FXS studies with a modest cohort size. However, the natural consistency in protein synthesis in control fibroblasts is not well understood. In synchronized MEFs, general rates of protein synthesis undergo a diurnal variation that is dependent on circadian regulators acting on mTORC1 components [81]. Translation is a key process involved in differentiation and cell fate specification and, recently, a slew of papers has identified key proteins that mediate this process [82,83]. Indeed, patterns of activation of mTORC1 change as cells differentiate [84,85]. Hence development and stem cell differentiation are multi-layered processes where key players governing translation itself are in constant flux.

In this state of dynamic equilibrium, loss of FMRP recreating FXS, creates a perturbation that has long term effects on cell fate and translation in general. Through chorionic villus sampling of human fetus, Willemsen et al. [86] demonstrated that FMRP loss occurs between 10–13 weeks in gestation. This is approximately when neuronal migration and maturation commences from neural stem cells. There is no clear in vivo study in rodent models to show deficits in this process in FXS. Similarly, there appears to no systematic cataloging of the changes in protein synthesis flux as ES or iPSC differentiate into neurons or glia and how this course changes in FXS derived cells. Without the support of this natural history, using translation as a biomarker or treatment yardstick will always be plagued by uncertainty. This is perhaps due to the fact that understanding the fluctuations in protein synthesis as a metabolic metric has frequently been overshadowed by the motivation to find what individual proteins are misregulated and hence offer potential node points for intervention.

Recently two studies were published that delved to some degree into matter changing transcriptomic hits with differentiation in FXS. Sunamara et al. [87] generated full knockouts (KO) of *FMR1* in hiPSC cells using CRISPR/Cas9 and transcriptomically characterized the differentiating cells from their isogenic controls. Loss of FMRP did not compromise the proliferation of the neural precursor cells derived from these cells. However, FMRP ablated neural precursor cells (NPCs) aberrantly expressed glial fibrillary acidic protein (GFAP) which continued even when the cells were differentiated into neurons. KO cells displayed transcriptomic profiles that were reminiscent of a more pluripotent stage rather than being committed to a neuronal or glial fate. Intriguingly though there was an increase in mTOR and S6 phosphorylation in hiPSCs lacking FMRP, which may allude to a higher translational rate. A second paper in 2018 by Richter laboratory [88] measured translational efficiency disruption in the event of FMRP loss in murine adult neural stem cells. Using ribosomal profiles and whole RNA sequencing, they were able to show that that FMRP loss affects different transcripts at different points of gene expression including translation, ribosome stalling and mRNA abundance. This suggests a possible dysregulation of translation in stem cells (human and murine-derived) and FMRP’s role in early development. Since stem cells are a population that are always in a state of dynamic equilibrium, there are likely to be effects on protein synthesis and causing variability in its measurement.

## 5. Patient Specific Treatment in FXS: Potential Ways to Leverage Protein Synthesis as a Diagnostic Marker

The importance of preclinical animal models in understanding the cardinal mechanisms underlying FXS and other models of autism spectrum disorders (ASD) cannot be overstated [89]. However, setbacks in the recent drug trials has perhaps had a positive outcome in highlighting the need for having interim validation of drug effects in patient-derived cells. Another transition, that has occurred, more for cancer therapeutics, is a move towards using patient-derived cells to tailor patient-specific treatments. An elegant demonstration of the power of this approach was Chia et al., [90] wherein cells isolated from cancer biopsy from the patient were used to identify the best therapy course. However, this work rested upon well-characterized and accepted yardsticks of cancer biology that are, at present, lacking for cultured patient-derived cells for FXS. 

This highlights a need to not only understand disease biology in a dish, but also to generate benchmarks in such systems that can be used for diagnostic purposes. One category of this could be signatures that individually or in combination, measure a cell-autonomous feature that can be reliably measured across multiple sites in adherence to an agreed-upon, consensus protocol. Structural and physiological signatures as mentioned above in neurons from FXS samples offer one such avenue. The downside is the long time and high cost involved in generating these neurons. A faster and cost-effective readout can be protein synthesis, which can give a quantum of holistic translation in a patient-derived fibroblast or LCL provided the background mutational load is known and the culture passage, media and incubation conditions are standardized. 

Imperatively, for therapeutic discovery or even therapy choice, it is prudent to appreciate that the cell types of the human brain do not function in isolation. It is a continuous orchestration of electrical and chemical signaling events organized in a spatio-temporal fashion in a tightly regulated microenvironment. It is increasingly becoming crucial to ask how each of these cell types communicate with the milieu around it. Clearly, this is lacking in the two-dimensional in vitro stem cell derived models and hence has been unable to mimic many of the diseased phenotypes [91]. Organoids are an initial step in this direction, wherein three-dimensional culturing of cells using suitable matrices, scaffolds and shearing force, has resulted in the capability to re-construct cerebral organoids [92]. These have been found to mimic neural development and incorporate structural and functional deficits in diseased samples [93,94]. Electrophysiological recordings from cerebral organoid slices [95] validates the functional properties of neurons from the different regions of the brain. Mariani et al. [96] has demonstrated that cerebral organoids derived from ASD patients show significant differences in the number of synaptic contacts made. Additionally, forebrain organoids from severe idiopathic ASD exhibit dysregulation of forkhead box G1 expression, high cell cycle progression and higher levels of GABA produced by the cells [96]. This is a simple example of how modelling using organoids can be used to identify new drugs using a candidate-based approach for studying aberrant neurodevelopmental processes. The key question would be whether protein synthesis fluxes in FXS organoids will be similar to those in 2-D cultured cells. 

To summarize, the FXS research community is slowly embracing the power of patient-derived cells for biomarker discovery. However with growing appreciation, there is a need to identify robust measures of disease-states that address with patient heterogeneity to predict accurately any treatment response. Protein synthesis is a consistently aberrant molecular phenotype in FXS, however there needs to be further research devoted to mapping the natural history of cells derived from FXS patients, controlled for intrinsic genetic variation and also experimental conditions. This is a fundamental characterization effort that needs to be undertaken by any outfit aiming to utilize patient-derived cells for developing diagnostics and therapeutic interventions.
